# Prenatal Lipid-Based Supplementation and Infant Weight-for-Length Z-score Outcomes in the First Year of Life: Evidence From a Community-Based Trial in Pakistan

**DOI:** 10.7759/cureus.99920

**Published:** 2025-12-23

**Authors:** Ijaz Habib, Sheraz Fazid, Mahamadou Tanimoune, Cecilia Garzon, Yasir Ihtesham, Fazal Dad, Zia Ul Haq

**Affiliations:** 1 Institute of Public Health and Social Sciences, Khyber Medical University, Peshawar, PAK; 2 Nutrition, United Nations, World Food Programme, Islamabad, PAK; 3 Nutrition, United Nations, World Food Programme, Peshawar, PAK; 4 Public Health, University of Glasgow, Glasgow, GBR

**Keywords:** antenatal care, benazir nashonuma programme, cluster-adjusted analysis, haz, iptw, lipid-based nutrient supplements, mq-lns, pakistan, wasting, wlz/whz

## Abstract

Background

The tribal belt of Pakistan continues to present the highest tendencies of acute malnutrition, a challenge commonly affecting children’s growth in infancy across South Asia. Whilst maternal lipid-based nutrient supplements have shown promising results, their effectiveness varies by formulation, dosage, and context. This study evaluated whether providing mothers with medium-quantity lipid-based nutrient supplements (MQ-LNS) during their pregnancy and six months postpartum could improve infant weight-for-length Z-scores (WLZs) at 12 months of age in a food-insecure district of Pakistan.

Methodology

This study used a community-based, non-randomized controlled trial design in Upper Kurram, Khyber Pakhtunkhwa, from February 2019 through January 2020. Cluster formation was based on Lady Health Worker catchment areas. The study provided Maamta MQ-LNS (75 g/day) to pregnant women from conception through six months of postpartum care. The control group received standard antenatal care (ANC) with iron-folic acid (IFA). The main study result consisted of WLZ measurements based on WHO standards at the 12-month follow-up. The study used multivariable linear regression with cluster-robust standard errors to establish effect sizes and inverse probability of treatment weighting to handle baseline differences. The research evaluated two additional outcomes through length-for-age z-score (HAZ) and wasting (WLZ < −2).

Results

The study results demonstrated that children whose mothers received MQ-LNS from pregnancy through the first half year of life gained better WLZ than control children at 12 months (adjusted β = 0.29; 95% confidence interval = 0.08-0.51; p = 0.007). The results showed no change in the difference after controlling for maternal and household variables. Mean HAZ was slightly higher in the intervention group (p = 0.038), while the proportion wasted (WLZ < −2) did not differ significantly between groups.

Conclusions

The research showed that rural mothers who consumed MQ-LNS during pregnancy and early postpartum delivered infants who had better WLZ at 12 months in a food-insecure setting. The findings showed a higher level of nutritional benefits than the standard prenatal small-quantity lipid-based nutrient supplement program, which indicates that MQ-LNS could provide additional benefit in cases of severe nutritional deficiencies. Policy integration within ANC and social protection initiatives, including the Benazir Nashonuma Programme, warrants consideration, alongside assessments of adherence, cost-effectiveness, and supply feasibility.

## Introduction

Undernutrition in children remains a leading cause of illness and death worldwide, contributing to nearly half of all deaths among children aged under five and leaving lasting effects on physical growth, cognitive development, and future productivity [1]. Globally, an estimated 45 million children are wasted, and 148 million are stunted, with South Asia bearing a disproportionate share of this burden [1]. The 2018 National Nutrition Survey of Pakistan reported that 7% of children under five years old were wasted and 40% were stunted, reflecting multiple causes, including inadequate maternal nutrition, poor diets, infection burden, and poverty [2,3]. Maternal undernutrition, including deficiencies in energy, protein, and micronutrients, remains a critical determinant of fetal growth restriction and postnatal faltering [3,4].

The first 1,000 days, from conception through a child’s second birthday, represent the most critical window for preventing malnutrition and its long-term consequences [3]. Yet, in many low-resource contexts, antenatal nutrition support remains limited, as programs typically provide only iron-folic acid (IFA) supplements [4]. Lipid-based nutrient supplements (LNS) were developed to bridge these nutrient gaps by offering energy, essential fatty acids, and multiple micronutrients in a convenient, ready-to-use form [5]. Two main formulations are in use: small-quantity LNS (SQ-LNS; 20 g per day) and medium-quantity LNS (MQ-LNS; 50-100 g per day) [6].

Evidence from prenatal SQ-LNS trials has shown modest yet consistent improvements in birth outcomes and early child growth, including reductions in severe wasting, though most studies to date have been conducted in sub-Saharan Africa [7-9]. Few studies from South Asia have assessed whether medium-quantity formulations during pregnancy and lactation improve infant weight-for-length z-scores (WLZs) within the first year of life [10]. While many publications report weight-for-height z-scores (WHZs) among older children, WLZ is the appropriate indicator of acute malnutrition below 24 months [11-13]. This highlights a regional evidence gap regarding locally adapted MQ-LNS and their effects on WLZ among food-insecure South Asian populations [14].

To address this gap, the World Food Programme (WFP) and the Government of Pakistan collaborated to develop Maamta, a locally produced MQ-LNS for pregnant and lactating women. Each 75 g daily ration provides approximately 350-400 kcal with balanced macronutrients and a full suite of micronutrients. Distribution was integrated into the Benazir Nashonuma Programme, providing supplements during pregnancy and up to six months postpartum through existing government service channels [15,16].

The biological rationale for MQ-LNS use is well established. Improved maternal nutrient reserves enhance placental transfer of fatty acids and micronutrients, promoting fetal tissue growth and higher birth weights [17]. Continued supplementation during early lactation increases breast-milk energy and micronutrient content, which supports faster infant growth and recovery from wasting [18]. Recent meta-analyses of prenatal SQ-LNS trials have demonstrated small but meaningful gains in WLZ and reduced wasting prevalence, underscoring their potential to improve early child growth [6]. However, differences between MQ-LNS and SQ-LNS in nutrient composition, energy density, and program delivery contexts underline the importance of locally generated evidence [19,20]. This is particularly relevant in settings where antenatal services remain narrowly focused on IFA supplementation, leaving wider maternal nutrient needs unmet [21,22].

The present study examines the effect of maternal MQ-LNS (Maamta) provided throughout pregnancy and the first six months postpartum on infant WLZ at 12 months compared with standard antenatal care (ANC), including IFA supplementation. The findings aim to inform policy and programmatic approaches for maternal nutrition support in Pakistan.

## Materials and methods

Study design and setting

**Figure 1:**
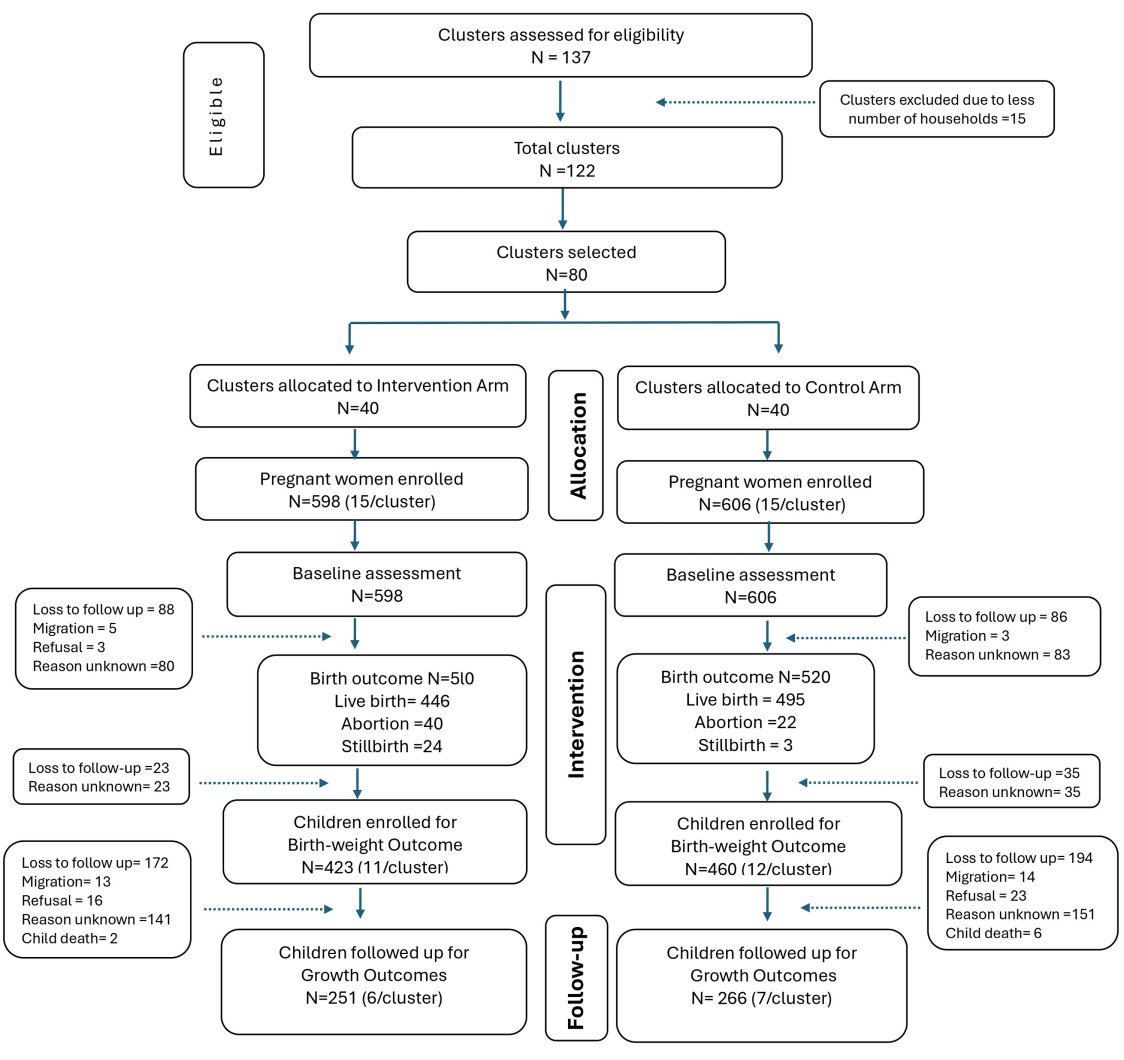
CONSORT flow diagram of cluster and participant progression. The figure shows cluster selection, enrolment of pregnant women, allocation to intervention and control arms, follow-up, and birth outcomes, as well as the final cohort of infants included in the analysis of weight-for-length z-score (WLZ) outcomes during the first year of life.

This was a community-based, non-randomized, controlled trial employing a two-stage cluster-sampling design conducted in Upper Kurram District, Khyber Pakhtunkhwa (Pakistan) between February 2019 and January 2020 (Figure 1 CONSORT flow diagram). The study aimed to evaluate the effects of Maamta, an MQ-LNS, on infant WLZ during the first year of life. Upper Kurram is a mountainous, food-insecure district where access to health and nutrition services is constrained, making it suitable for evaluating maternal nutrition interventions within routine service-delivery platforms [1]. This trial was prospectively registered with the International Standard Randomised Controlled Trial Number (ISRCTN) Registry (ID: ISRCTN94319790) on December 11, 2017.

Sample size determination

The sample size was estimated using both cluster- and individual-level calculations. The design effect (DE) was computed using the standard formula DE = [(n − 1)ρ + 1], where n = 15 (average cluster size) and ρ = 0.05 (intra-cluster correlation), resulting in a DE of 1.7 [23]. For an individually allocated trial with a β of 0.20, α of 0.05, effect size of 0.21, and SD of 1.0, the required effective sample was 705 participants. Adjusting for the design effect and average cluster size yielded an estimated 80 clusters.

Separately, a single-proportion approach (Z = 1.96, p = 0.40, d = 0.04) produced an initial estimate of n₀ = 576, which, after applying a DE of 2.0 and accounting for a 5% non-response rate, resulted in a final target sample of 1,210 participants. Ultimately, 1,209 first-trimester pregnant women were enrolled through household visits using Lady Health Worker (LHW) registers. The cluster size and target population were further validated using the University of Aberdeen cluster-trial calculator [24].

Sampling and participants

A total of 80 clusters were selected randomly from a sampling frame of 122 eligible clusters using a two-stage cluster-sampling technique. Each cluster corresponded to the catchment area of an LHW serving approximately 100-150 households. Clusters where households were officially assigned to LHWs implementing the Maamta programme were designated as intervention clusters, while control clusters were geographically comparable LHW catchments providing routine ANC, including IFA supplementation but without Maamta distribution. Eligible participants were pregnant women in their first or second trimester, residing permanently in the study area, intending to remain there for at least one year, and providing written informed consent. Exclusion criteria included multiple pregnancies, severe maternal illness, and any known congenital anomaly or condition likely to affect fetal growth [2].

Intervention

Participants in the intervention arm received Maamta, a locally produced MQ-LNS developed by WFP and the Government of Pakistan for pregnant and lactating women. Each daily ration provided 75 g (~350-400 kcal) of energy with balanced protein, essential fatty acids, and multiple micronutrients designed to complement local diets. Maamta was distributed monthly, starting at pregnancy enrolment (preferably first trimester) and continued through six months postpartum. Distribution occurred via health-facility delivery points and home visits by LHWs and nutrition assistants, who also reinforced dietary counseling, compliance checks, and hygienic storage [3].

Control

Women in the control clusters received routine ANC through government facilities, including standard IFA supplementation as per national guidelines (60 mg iron + 400 μg folic acid daily), counseling, and referral support, but no LNS products. Both study arms had access to the same primary healthcare infrastructure and maternal-child health services to ensure comparability [4].

Outcome measures

The primary outcome was infant WLZ at 12 months of age, calculated using the WHO Child Growth Standards (2006) [25]. Infant length and weight were measured to the nearest 1 mm and 0.1 kg, respectively, using standardized SECA 417 infantometers and digital SECA scales. The secondary outcome was length-for-age z-score (HAZ). Anthropometric data were collected by trained enumerators following WHO procedures, with daily calibration of equipment and duplicate measurements to ensure reliability. This approach aligns with recent national evidence on maternal nutrition and dietary diversity in Pakistan [26].

Data collection and quality assurance

Structured questionnaires captured sociodemographic, dietary, and obstetric information at enrolment. Field teams received intensive training on interviewing, anthropometry, and data quality control. Supervisors conducted random spot-checks and re-measurements on 10% of participants. Data were double-entered and validated using EpiData v3.1 before export to Stata version 14 (StataCorp., College Station, TX, USA) for analysis [6].

Statistical analysis

Baseline characteristics were summarized using means ± SDs for continuous variables and frequencies (%) for categorical variables. Independent t-tests and chi-square tests compared group differences at baseline and follow-up. The effect of the intervention on WLZ and HAZ was estimated using multivariable linear regression models adjusting for maternal age, education, parity, socioeconomic status, and infant sex. Because the design was cluster-controlled, all analyses applied cluster-robust standard errors to account for intra-cluster correlation. To further minimize selection bias, inverse probability of treatment weighting (IPTW) based on propensity scores was used, generating stabilized weights for covariate balance [8,16]. Statistical significance was set at p-values <0.05.

## Results

Participant flow and follow-up

A total of 80 clusters were enrolled in the study, representing the catchment areas of LHWs in Upper Kurram District. All eligible pregnant women identified within selected clusters were invited to participate. Overall, 517 mother-infant pairs had complete WLZ data at 12 months and were included in the final analysis, comprising 251 in the intervention group and 266 in the control group. Follow-up was high across both arms, with minimal loss to follow-up, and no significant differential attrition was observed between groups.

Primary outcome

Weight-for-Length Z-Score

**Figure 2:**
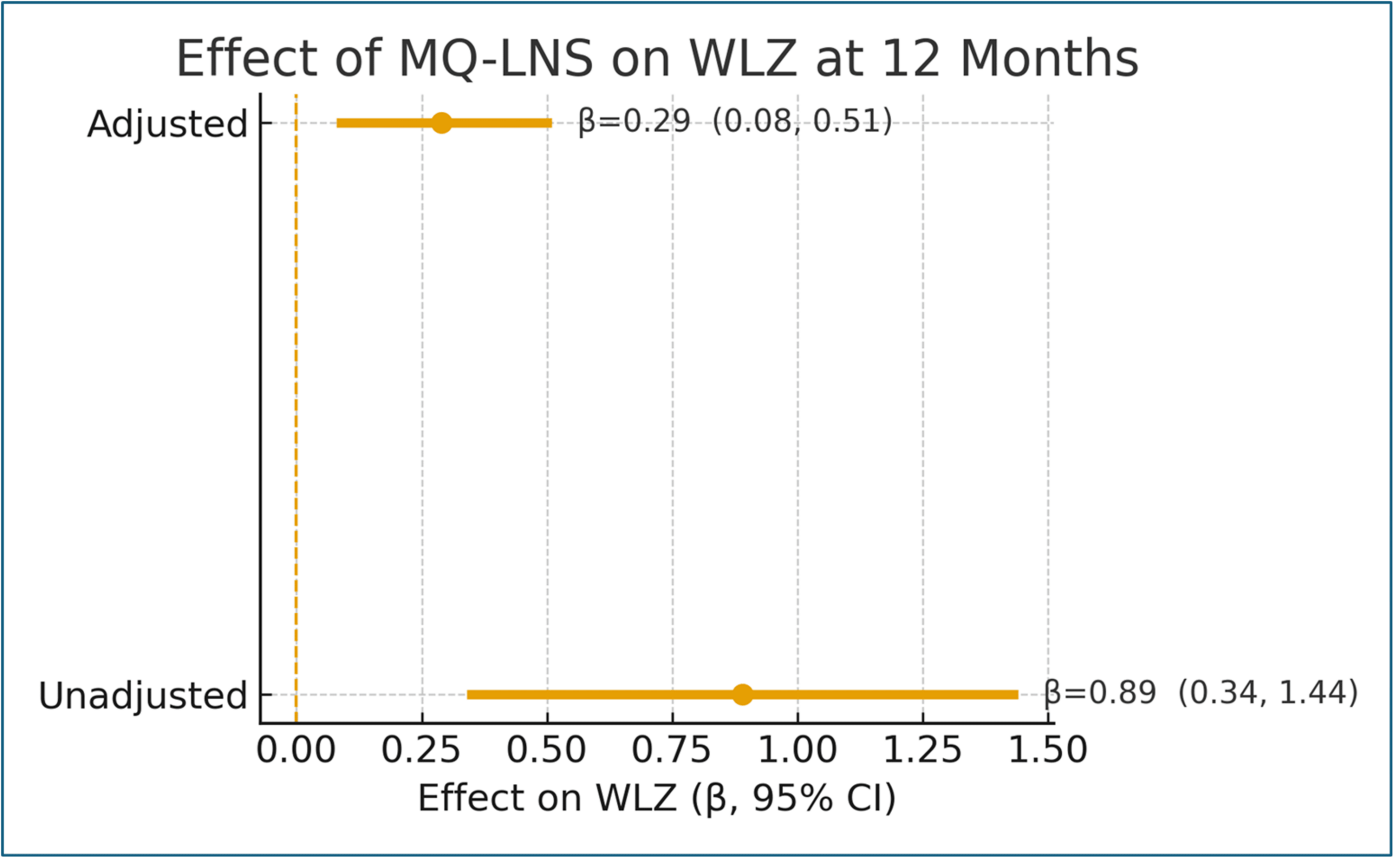
Error bar chart illustrating the unadjusted and adjusted associations between MQ-LNS exposure and infant WLZ at 12 months (β estimates with 95% CI). MQ-LNS = medium-quantity lipid-based nutrient supplement; WLZ = weight-for-length Z-score; CI = confidence interval

Maternal supplementation with MQ-LNS during pregnancy and for six months postpartum was associated with a statistically significant improvement in infant WLZ at 12 months (Table 1, Figure 2). After adjustment for clustering and key covariates, infants in the intervention group had significantly higher WLZ compared with controls (β = 0.29, 95% confidence interval (CI) = 0.08 to 0.51; p = 0.007). This effect remained robust in models adjusting for potential confounders, and the positive association persisted when using IPTW to account for baseline imbalances between groups. The magnitude of improvement represents a clinically meaningful benefit for both individual children and at the population level, with potential implications for reducing the risk of early childhood wasting.

**Table 1:** WLZ outcomes (unadjusted and adjusted models). Notes: Multivariable linear regression adjusted for infant age and sex, maternal education, household food security, wealth index, and birthweight; cluster-robust SE used. IPTW models yielded directionally consistent results. Bold indicates p < 0.05. WLZ = weight-for-length Z-score; IPTW = inverse probability of treatment weighting; CI = confidence interval

Model	β (coefficient)	95% CI	P-value
Unadjusted	0.89	0.34, 1.44	0.004
Adjusted	0.29	0.08, 0.51	0.007

Baseline Characteristics

Baseline maternal and household characteristics were generally comparable between study arms (Table 2), following cluster adjustment. Maternal age, parity, education, household size, and socioeconomic indicators showed no meaningful differences between intervention and control groups. Infant sex distribution at birth was also similar. These findings indicate balanced baseline profiles, supporting the internal validity of subsequent group comparisons.

**Table 2:** Baseline characteristics of participants (n = 517). Notes: Values are means ± standard deviations or percentages with corresponding n-values. Test statistics are derived from independent t-tests (continuous variables) and chi-square tests (categorical variables). No statistically significant differences were observed at baseline.

Characteristic	Intervention (n = 251)	Control (n = 266)	Test statistic	P-value
Maternal age (years), mean ± SD	26.8 ± 5.3	27.2 ± 5.7	t = 0.77	0.442
No formal education	44.6% (112/251)	46.3% (123/266)	χ² = 0.17	0.682
Parity, mean ± SD	3.4 ± 1.7	3.6 ± 1.8	t = 1.00	0.317
Household food insecurity	61.8% (155/251)	63.1% (168/266)	χ² = 0.10	0.745
Wealth index (poor)	52.9% (133/251)	54.5% (145/266)	χ² = 0.18	0.678
Infant sex (male)	50.2% (126/251)	49.8% (132/266)	χ² = 0.01	0.924
Low birthweight (<2.5 kg)	12.7% (32/251)	13.2% (35/266)	χ² = 0.03	0.871

Secondary outcome

Length-for-Age Z-Score

No meaningful difference in mean HAZ at 12 months was observed between study groups (Table 3). Although the intervention group demonstrated a slightly higher average HAZ, the difference was small and not clinically significant. This is consistent with global evidence demonstrating that linear growth (represented by HAZ) is less responsive to single-nutrient or product-based interventions within the first year of life and generally requires longer-term, multisectoral improvements to shift growth trajectories. Therefore, while MQ-LNS supplementation contributed to improvements in WLZ, it did not produce a measurable impact on linear growth by 12 months.

**Table 3:** HAZ at 12 months by intervention status (n = 517). Notes: Independent two-sample t-test used to compare group means. HAZ = length-for-age z-score

Variable	Mean HAZ ± SD	95% CI	t-score	P-value
Intervention (MQ-LNS)	-0.84 ± 1.12	-1.01 to -0.67	t = 2.08	0.038
Control	-1.03 ± 1.14	-1.21 to -0.86	Reference	—

Wasting Prevalence

Analysis of wasting prevalence (WLZ < -2) at 12 months showed no statistically significant difference between groups (Table 4). While the prevalence of wasting was lower in the intervention arm relative to the control arm, the difference did not reach statistical significance. These findings suggest that although MQ-LNS effectively improved WLZ as a continuous measure, a larger sample or longer duration may be required to detect significant reductions in categorical wasting prevalence.

**Table 4:** Wasting (WLZ < -2) at 12 months. Notes: Linear probability (RD) and Poisson regression (RR) adjusted for covariates as per Table 2; robust SE applied. WLZ = weight-for-length Z-score; CI = confidence interval

Metric	Estimate	95% CI	P-value
Adjusted risk difference (pp)	1.35	-	0.515
Adjusted risk ratio	1.27	0.63, 2.53	0.503

Sensitivity and robustness analyses

To strengthen confidence in the observed WLZ effect, multiple sensitivity analyses were conducted. Cluster-adjusted models yielded consistent effect estimates, confirming that the intervention effect was not driven by cluster-level variation. IPTW models further demonstrated that the WLZ improvement remained stable after balancing key maternal and household characteristics across study arms. The convergence of results across unadjusted, adjusted, and weighted models provides evidence that the WLZ benefit observed in the MQ-LNS group is robust to analytical approach and unlikely to be explained by confounding.

## Discussion

The research indicated that mothers who consumed locally produced MQ-LNS during pregnancy and postpartum achieved significantly higher infant weight gain at 12 months (adjusted β = 0.29; 95% CI = 0.08-0.51; p = 0.007). The cluster-adjusted and IPTW analyses confirmed the robustness of this intervention effect. The observed magnitude was larger than the pooled WLZ improvements typically reported in multi-country meta-analyses of prenatal SQ-LNS supplementation (0.05-0.20 SD) [5,6], suggesting that MQ-LNS may offer greater benefits for food-insecure South Asian populations [20].

The results align with findings from previous studies in Ghana, Malawi, and Bangladesh, which demonstrated the positive influence of LNS on birth outcomes and infant development, though effect sizes varied by setting [7-9]. The greater WLZ gains in the present study may be attributed to the higher energy and micronutrient content of MQ-LNS compared with SQ-LNS, as well as to extended supplementation for six months postpartum, which likely improved breast milk quality.

Continuous support from LHWs and strong community engagement within the culturally cohesive district of Kurram further enhanced adherence. Collectively, these contextual factors likely contributed to the observed outcomes. These findings are also consistent with earlier analyses conducted within the same trial framework [10]. While WLZ outcomes improved significantly, no substantial effects were detected on HAZ at the 12-month follow-up. This aligns with evidence suggesting that improvements in linear growth require longer exposure periods and complementary investments in WASH, infection control, and poverty reduction interventions [11-13]. It is also important to note that this study was not powered primarily to detect differences in HAZ, which limits inferences regarding linear growth.

The study benefits from real-world program delivery, which enhances external validity. It also employed cluster-robust analyses and IPTW methods to minimize selection bias, while routine LHW follow-ups supported adherence and ensured standardized anthropometric measurements based on WHO protocols [14-16]. However, as a non-randomized cluster study, some residual confounding may persist despite adjustments and weighting procedures [15,16]. Additional limitations include reliance on self-reported adherence data and a single 12-month follow-up, which restricts the interpretation of WLZ trajectories over time. Furthermore, the study lacked sufficient power to assess HAZ or explore morbidity pathways. Despite these limitations, the results are highly applicable to food-insecure rural contexts in Pakistan, though replication in other provinces and service platforms is warranted.

From a policy perspective, integrating maternal MQ-LNS into routine antenatal and postnatal care could meaningfully reduce early wasting in high-burden regions. The Benazir Nashonuma Programme and provincial health systems could leverage these findings to strengthen service delivery, provided that cost-effectiveness, supply chain reliability, and quality assurance are ensured [17-19]. Incorporating MQ-LNS into maternal care pathways may thus contribute to addressing Pakistan’s persistent challenge of acute malnutrition when supported by effective monitoring systems [20,21].

Future research will extend to dose-response and adherence analyses, cost-effectiveness evaluations, and longer-term follow-up beyond 12 months to capture linear growth and neurodevelopmental outcomes. Further integration with WASH and social protection initiatives will be explored to address multiple determinants of undernutrition [11-13,22]. Complementary qualitative research on cultural acceptability will also guide context-specific expansion strategies.

In summary, the study demonstrates that maternal MQ-LNS consumption during pregnancy and early lactation supports improved infant weight gain at 12 months in food-insecure Pakistani communities. The substantial effect size observed exceeds that typically reported for SQ-LNS, reinforcing the value of integrating maternal supplementation into antenatal and postnatal nutrition programs in similar settings.

## Conclusions

This community-based controlled trial in Upper Kurram, Pakistan, showed that maternal MQ-LNS supplementation during pregnancy and the first six months after birth resulted in a substantial WLZ improvement at 12 months (adjusted β = 0.29; 95% CI = 0.08-0.51), and the effect remained consistent when cluster-adjusted and IPTW models were used. The HAZ measurements at 12 months showed no significant changes, which supports the requirement for extended exposure and multiple-sector involvement to produce linear growth changes. The intervention group produced less waste, but the results did not achieve statistical significance because more participants and a longer study duration would be required to detect categorical variations. The implementation of maternal MQ-LNS in food-insecure areas leads to better ponderal growth in infants during their first year of life, which results in lower wasting rates at a population level. ANC platforms in Pakistan and the Benazir Nashonuma Programme with supply chain management and cost-effective adherence support will help accelerate the reduction of infant acute malnutrition. The success of the Benazir Nashonuma Programme in Pakistan is acknowledged globally, and replications are underway in other low- and middle-income countries.
